# Enhancing Cytotoxicity of Tamoxifen Using *Geranium* Oil

**DOI:** 10.1155/2022/8091339

**Published:** 2022-03-16

**Authors:** Dai Mizuno, Masahiro Kawahara, Keiko Konoha-Mizuno, Kentaro Yamazaki

**Affiliations:** ^1^Department of Forensic Medicine, Faculty of Medicine, Yamagata University, 2-2-2 Iida-Nishi, Yamagata-shi, Yamagata 990-9585, Japan; ^2^Research Institute of Pharmaceutical Sciences, Faculty of Pharmacy, Musashino University, 1-1-20 Shin-Machi, Nishitokyo-shi, Tokyo 202-8585, Japan

## Abstract

Aromatherapy and plant-based essential oils are widely used as complementary and alternative therapies for various symptoms, including anxiety, mild mood disorders, and cancer-related pain. In a previous study, we developed an in vitro assay using immortalized hypothalamic neuronal cells (GT1-7 cells). In this study, we used this assay to investigate the effects of *Geranium* oil on the cytotoxicity of the oestrogen receptor (ER) antagonist: tamoxifen (TMX). The results showed that *Geranium* oil augmented TMX-induced cell death in a dose-dependent manner without directly reducing the viability of GT1-7 cells. Cotreatment with *Geranium* oil and ER agonist *β*-estradiol (E2) attenuated the inhibition of GT1-7 cell growth. Moreover, *Geranium* oil and geraniol, a major constituent of *Geranium* oil, showed weak agonist activity on ER*α* and ER*β* with geraniol augmenting TMX-induced cell death similar to that observed in *Geranium* oil. Both compounds impair E2 activity. These data indicate that geraniol is an essential constituent of *Geranium* oil.

## 1. Introduction

Trace nutrients, such as vitamins and minerals, contained in plants are essential for human health. The essential oils used in aromatherapy are good for health. Aromatherapy is a folk remedy that encompasses the use of essential oils derived from various types of plant sources for a variety of applications and is widely used as a complementary or alternative therapy for symptoms including anxiety and depression [1].

Aromatherapy is performed by irritation to olfaction, or by application, inhalation, or oral administration of essential oils with the aroma component of plants and fruits. The chemical components in the essential oils bind with the receptors in the olfactory bulb causing these effects. They stimulate the limbic system (the emotional center of the brain) which affect the physiological functions of the autonomic nervous, endocrine, and immune systems via hormones, neurotransmitters, and cytokines. Fragrant compounds and essential oils with sedative effects have influenced the motility of mice in inhalation studies [2]. Another report indicated that the odour of lavender ameliorated the influence of stress on brain mechanisms involved with the circadian rhythm of the autonomic nervous system activities and maintains their activity pattern during sleep [3]. Moreover, bergamot might be useful as an aromatherapeutic to minimise the symptoms of stress-induced anxiety, mild mood disorders, and cancer-related pain [4]. There is a movement to complement the missing parts of Western medicine by using aromatherapy to improve medical methods (medical aromatherapy), with potential applications in obstetrics and gynaecological diseases, skin diseases, psychosomatic diseases, pain management, stress management, and upper respiratory tract infection treatment [1, 2, 5–7].

The olfactory system not only transmits odour information but also acts as an entry pathway for outside substances. Molecules can be transported from the olfactory epithelium to the olfactory bulb via retrograde axonal transport. Kanayama et al. [8] reported that metal ions administered intranasally are transported from the nasal cavity to the olfactory bulb via the olfactory nerve pathway and from there to other brain regions such as the hypothalamus and hippocampus. Another study found significant levels of fragrant compounds in the plasma after inhalation, indicating that the effects of aromatherapy result from direct pharmacological interaction rather than indirect central nervous system relays [2]. The components of essential oils applied to the skin using an aromatic bath or massage can be absorbed through the epidermis, dermis, hair follicles, and sebaceous glands. In cases of inhalation or oral administration, oils absorbed from mucous membranes in the nasal cavities and the gastrointestinal tract enter the blood and lymph vessels to potentially exert effects on the biological systems, such as the nervous system [1, 2].

We previously showed that direct pharmacological interactions of several essential oils could be investigated using GT1-7 cell lines developed by genetically targeting the tumorigenesis of mouse hypothalamic neurons [9]. We found in a previous study that *Geranium* oil increased GT1-7 cell death induced by the oestrogen receptor (ER) antagonist tamoxifen (TMX) [9]. *Geranium* oil is commonly used as an element in aromatherapy owing to its many health benefits. It is used as a holistic treatment to improve physical, mental, and emotional health and has potential applications as an antidiabetic, anticancer, antibacterial, and antimicrobial agent [1]. Nakamura et al. reported that *Geranium* oil might be useful in ameliorating symptoms linked to premenstrual syndrome (PMS) [10]. *β*-Estradiol (E2) is involved in the regulation of gonadotropin-releasing hormone (GnRH) secretion in conjunction with ER. *Geranium* oil may affect GnRH secretion through the ER, contributing to the moderating effect of this oil on PMS. In this study, we investigated the components and mechanisms underlying the elevation of TMX-induced cell death in GT1-7 cells following the administration of *Geranium* oil. Essential oil is a mixture of various organic compounds and the effects of essential oils are likely due to the synergistic or competitive actions of these compounds, which need to be identified.

## 2. Materials and Methods

### 2.1. Reagents

*Geranium* oil was purchased from Tree of Life Co., Ltd. (Tokyo, Japan), TMX was purchased from Sigma-Aldrich (St. Louis, MO, USA), geranyl acetate was purchased from Tokyo Chemical Industry Co., Ltd. (Tokyo, Japan), and other reagents were purchased from FUJIFILM Wako Pure Chemical Co., Ltd. (Osaka, Japan). These compounds were diluted in dimethyl sulfoxide (DMSO) prior to use.

### 2.2. Cell Culture

GT1-7 cells (provided by Dr. R. Weiner, University of California at San Francisco, CA, USA) were grown in Dulbecco's modified Eagle's medium/nutrient mixture F-12 Ham (DMEM/F-12) supplemented with 10% foetal bovine serum. After enzymatic digestion using trypsin, the cells were suspended in a serum-free medium and plated onto culture plates [11]. The cells were then cultured in a humidified incubator at 37°C and 5% CO_2_.

### 2.3. Cell-Viability Assay

Cell viability was assessed as described previously [12]. Briefly, dissociated GT1-7 cells were plated onto 96-well culture plates at a concentration of 5 × 10^4^ cells/well in 200 *μ*L of culture medium. Following 24 h incubation, the cells were treated with *Geranium* oil at a final concentration of 5 *μ*g/mL, 10 *μ*g/mL, and 25 *μ*g/mL, respectively. The major constituents of *Geranium* oil were used at the same concentration in the absence and presence of 5 × 10^−8^ M E2. Then, 0.5 *μ*M TMX was immediately added to the medium. The final concentrations of the major constituents of *Geranium* oil are listed in Table 1. After 24 h of exposure, cell viability was quantified using the WST-1 assay with the Cell Counting Kit (Dojindo, Kumamoto, Japan). The absorbance of the treated samples was measured against a blank control using a microplate reader (iMark™ microplate absorbance reader; Bio-Rad Laboratories, Hercules, CA, USA). The detection and reference wavelengths were 450 nm and 620 nm, respectively.

### 2.4. Analysis of the Components of *Geranium* Oil

The major components of *Geranium* oil were analysed using a G1888 static headspace sampler connected to an Agilent 7890 gas chromatography (GC) system (Agilent Technologies, Mississauga, Ontario, Canada). GC analysis was performed according to the manufacturer's instructions, with minor modifications. The 20 mL headspace vials and aluminium crimp vial caps were purchased from Agilent Technologies. *Geranium* oil or its components (Table 1) with ethyl salicylate (internal standard) were diluted to 200 *μ*L using hexane and encapsulated in 20 ml vials for headspace experiments. The experiment was conducted under the following conditions: oven temperature: 80°C; loop temperature: 90°C; transfer line: 130°C; equilibration time: 30 min; shaking speed: high; pressurization time: 0.15 min; carrier gas: He; in-vial pressure: 103421 Pa; loop fill: 0.15; loop equilibration: 0.05; and injection time: 1.00. GC analysis was performed on an Agilent 7890 GC equipped with a flame ionisation detector (FID) and a capillary column (DB-WAX; 20 m × 0.18 mm, 0.18 *μ*m film thickness). The carrier gas (He) was administered at a flow rate of 1.33 mL/min, the oven temperature program ranged from 45–250°C at 7.79°C/min, and the FID and transfer-line temperatures were set to 250°C. The relative percentages of the compounds were calculated based on the peak areas from the FID data.

### 2.5. Nuclear Receptor Cofactor Assays

The ER*α*/*β* agonist activity of *Geranium* oil or TMX was monitored using EnBio receptor cofactor assay system (RCAS) for ER*α* and ER*β* kits (Fujikura Kasei Co., Ltd., Ibaraki, Japan) according to the manufacturer's instructions. We added 95 *μ*L of ER*α* or ER*β* solution to each coactivator (SRC1)-immobilised well. Then, 5 *μ*L *Geranium* oil (final concentrations: 10–25 *μ*g/mL), geraniol (final concentrations are shown in Table 1), or (±) citronellol (final concentrations are shown in Table 1) was added in the absence and presence of E2 (final concentration: 1 nM) and TMX (final concentration: 25 *μ*M). As a positive control, we added 5 *μ*L of the 20 nM E2 and DMSO mixture to each appropriate well after adding the 95 *μ*L ER solution. The plate was then incubated at room temperature for 1 h while shaking (∼800 rpm). All the wells were aspirated and washed with wash buffer included in the kit (three times). Then, a 100 *μ*M detection antibody solution was added to each well and incubated at room temperature for 30 min while shaking (∼800 rpm). All wells were again aspirated and washed (three times). Then, the 100 *μ*M TMB substrate was added to all the wells and incubated at room temperature for 20 min, after which a 100 *μ*M stop solution was added to all the wells. The chromogen produced was measured at an absorbance of 450 nm using an iMark™ microplate absorbance reader (Bio-Rad Laboratories).

### 2.6. Statistical Analyses

All statistical evaluations were performed using a two-tailed student's *t*-test with the KareidaGraph v 4.0 Software (Synergy Software, PA, USA). A probability level (*p*) of <0.01 or <0.05 was considered significant.

## 3. Results

### 3.1. *Geranium* Oil Enhances TMX Cytotoxicity

**Figure 1:**
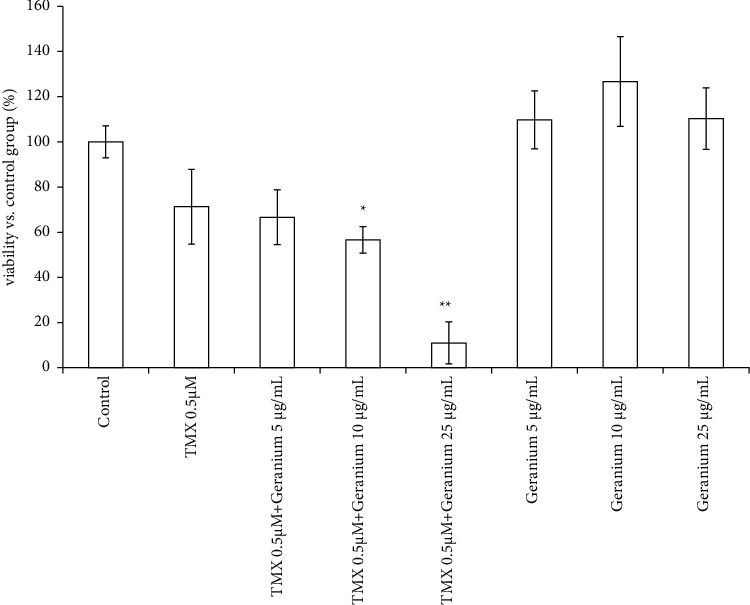
*Geranium* oil enhances TMX cytotoxicity. GT1-7 cells were treated with 0.5 *μ*M TMX in the presence and absence of various concentrations of *Geranium* oil. After 24 h, the cell viability was analysed using WST-1 assay. Data represent the mean ± standard error of the mean (*n* = 6). ^*∗*^*p* < 0.05, ^*∗∗*^*p* < 0.01 vs. the TMX 0.5 *μ*M group.

The GT1-7 cell line is a useful model for investigating endocrine-disrupting chemicals, such as TMX [13]. In this, we investigated the effect of *Geranium* oil on TMX-induced cell death in GT1-7 cells (Figure 1). Treatment with 0.5 *μ*M TMX significantly decreased the cell viability of the GT1-7 cells to 71.3 ± 16.5% (*p* < 0.01 vs. the control group). Administration of *Geranium* oil augmented TMX-induced cytotoxicity in a dose-dependent manner. Treatment with 5 *μ*g/mL, 10 *μ*g/mL, and 25 *μ*g/mL *Geranium* oil along with 0.5 *μ*M TMX decreased the cell viability by 66.6 ± 12.1%, 56.6 ± 5.9% (*p* < 0.05 vs. the TMX 0.5 *μ*M group), and 11.0 ± 9.3% (*p* < 0.01 vs. the TMX 0.5 *μ*M group), respectively (Figure 1). There was no significant difference between the cell viabilities of the control and *Geranium* oil-treated groups (Figure 1). These results showed that *Geranium* oil augmented TMX-induced cytotoxicity without directly reducing the cell viability of GT1-7 cells.

### 3.2. *Geranium* Oil Displays Weak Agonist Activity on Both ER*α* and ER*β*

**Figure 2:**
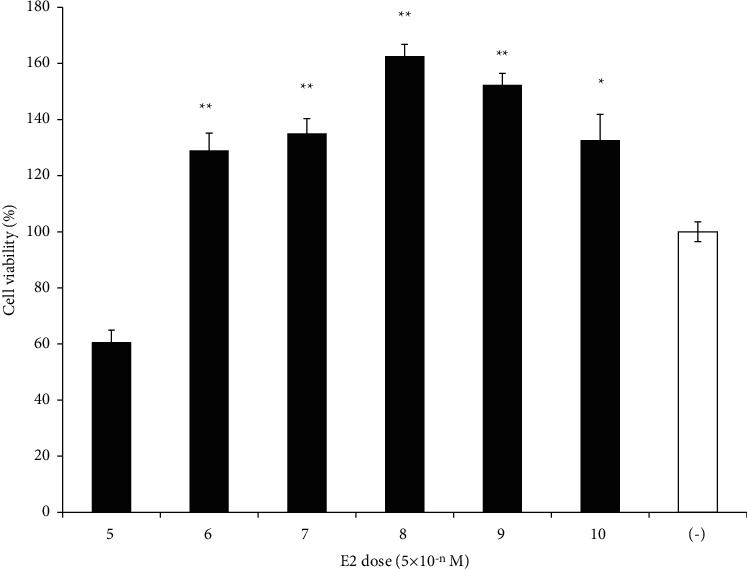
Dose response of E2 with respect to the viability of GT1-7 cells. GT1-7 cells were treated with various concentrations (5 × 10^−10^ to 5 × 10^−5^ M) of E2. After 24 h, the cell viability was analysed using WST-1 assay. The *x* axis represented the dose of E2. Data represent the mean ± standard error of the mean (*n* = 6). ^*∗*^*p* < 0.05, ^*∗∗*^*p* < 0.01 vs. the E2 free group (white graph).

**Figure 3:**
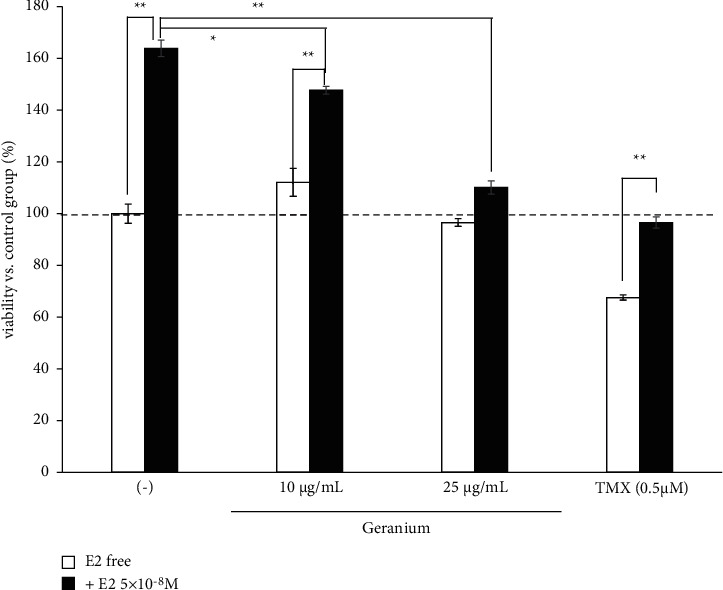
Effects of TMX and *Geranium* oil on GT1-7 cell viability. Cells were treated with 0.5 *μ*M TMX or various concentrations of *Geranium* oil and cultured in the presence and absence of E2 (5 × 10^−8^ M). After 24 h, the cell viability was analysed using WST-1 assay. Data represent the mean ± standard error of the mean (*n* = 6). ^*∗*^*p* < 0.05, ^*∗∗*^*p* < 0.01.

To investigate the involvement of the ER in the enhancement of TMX-induced cell death, we administered *Geranium* oil and/or TMX to GT1-7 cells in the presence and absence of 5 × 10^−8^ M E2. The results showed that E2 increased the viability of the GT1-7 cells in a dose-dependent manner, with 5 × 10^−8^ M E2 significantly increasing the cell viability by 162.6 ± 4.2% (maximum) (Figure 2). Moreover, E2 significantly inhibited TMX-induced cell death. Treatment with 5 × 10^−8^ M E2 almost inhibited cell death induced by the 0.5 *μ*M TMX (Figure 3). Interestingly, coadministration of *Geranium* oil with E2 significantly inhibited GT1-7 cell growth induced by E2 (Figure 3).

**Figure 4:**
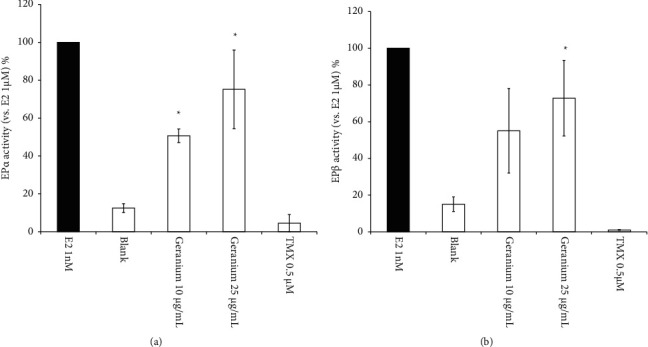
ER agonist activity of *Geranium* oil. *Geranium* oil showed weak agonist activity on (a) ER*α* and (b) ER*β*. Interactions between ER*α*/*β* and *Geranium* oil (final concentrations: 10 and 25 *μ*g/mL) and/or TMX (final concentration: 0.5 *μ*M) were monitored using RCAS for ER*α* and ER*β* kits according to the manufacturer instructions. The black graph represents ER agonist activity of positive control (1 nM E2). Data represent the mean ± standard error of the mean (*n* = 3). ^*∗*^*p* < 0.05 vs. the blank group.

The RCAS indicated that *Geranium* oil displayed weak agonist activity on both ER*α* and ER*β*, with this activity increasing in a dose-dependent manner (Figures 4(a) and 4(b)). In contrast, TMX did not display ER agonist activity. Thus, *Geranium* oil showed weak agonist activity on both ER*α* and ER*β*.

### 3.3. Quantitative Analysis of *Geranium* Oil Components by Gas Chromatograph

**Figure 5:**
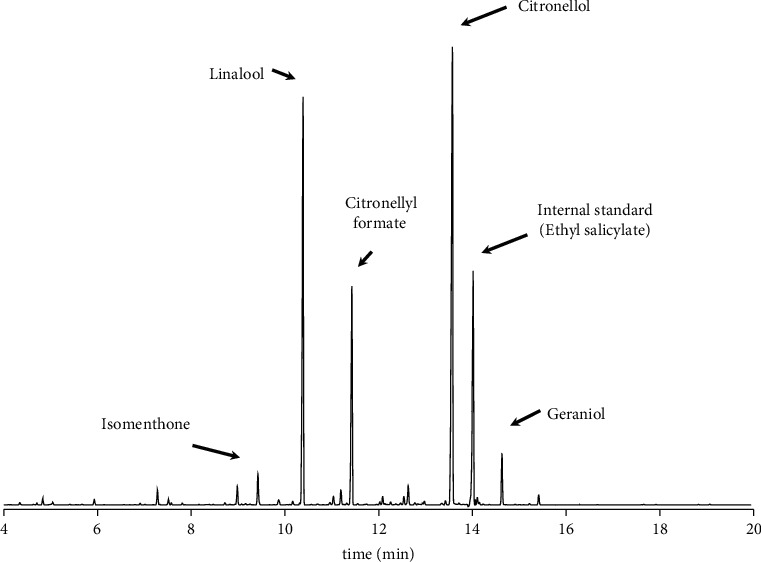
Chromatograms of major components of *Geranium* oil. The major components of *Geranium* oil were analysed using a G1888 static headspace sampler connected to an Agilent 7890 GC system. *Geranium* oil and its components are listed in Table 1, along with ethyl salicylate as an internal standard. Retention times and component concentrations were evaluated using commercial reagents as standards.

Various compounds are present in *Geranium* oil. In particular, citronellol, linalool, citronellyl formate, geraniol, and isomentone are present in high concentrations [14]. In this study, we quantified these components using GC. We detected peak derived from each compound according to their respective retention times (9.0 min for isomentone, 10.4 min for linalool, 11.4 min for citronellyl formate, 13.6 min for citronellol, and 14.6 min for geraniol); ethyl salicylate (14.0 min) was used as an internal standard. Calibration curves were generated for each compound to determine their respective concentration in the *Geranium* oil (Figure 5 and Table 1).

### 3.4. Geraniol Enhancing Effect of *Geranium* Oil on TMX Cytotoxicity

**Figure 6:**
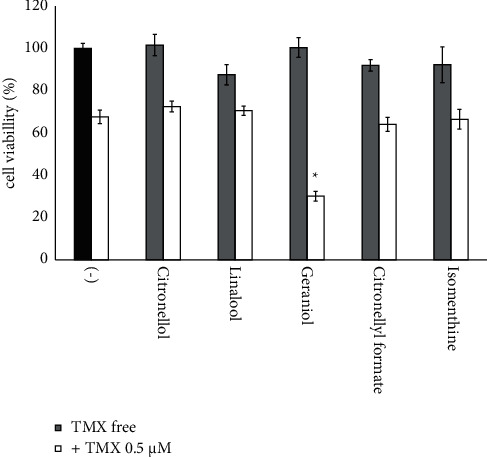
Effects of *Geranium* oil compounds on TMX cytotoxicity. GT1-7 cells were treated with 0.5 *μ*M TMX in the presence and absence of different concentrations of compounds equivalent to 25 *μ*g/mL of *Geranium* oil (table). After 24 h, the cell viability was analysed using WST-1 assay. Data represent the mean ± standard error of the mean (*n* = 6). The black graph represents the data of the untreated group. ^*∗*^*p* < 0.01 vs. the (−) + TMX (0.5 *μ*M) group (white graph in the (−) group).

To clarify the mechanisms involved in the enhancement of TMX-induced cytotoxicity by *Geranium* oil, we investigated the effects of the respective constituents of *Geranium* oil identified using GC analysis on TMX-induced (0.5 *μ*M) cell death. Using concentrations of each component equivalent to that contained in 25 *μ*g/mL *Geranium* essential oil solution, we found that none of the components significantly altered the viability of GT1-7 cells in the absence of TMX relative to the control group (black and gray graph in Figure 6). When coadministered with 0.5 *μ*M TMX, geraniol significantly reduced the cell viability from 67.6 ± 3.1% to 30.1 ± 2.3% as compared to TMX alone (white graph in Figure 6). None of the other components showed a significant effect on TMX-induced cytotoxicity. These data suggest that geraniol is essential for the effect of *Geranium* oil on TMX-induced cytotoxicity in GT1-7 cells.

**Figure 7:**
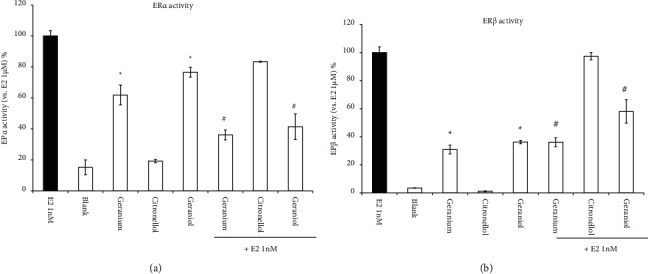
Geraniol attenuates ER-agonist activities. Geraniol attenuates (a) ER*α*- and (b) ER*β*-agonist activities. Interactions between ER*α*/*β* and geraniol or citronellol at final concentrations equivalent to 25 *μ*g/mL of *Geranium* oil (Table 1) or *Geranium* oil (final concentration: 25 *μ*g/mL) were monitored using the RCAS for ER*α* and ER*β* in the absence or presence of E2 (final concentration: 1 nM). The black graph represents ER agonist activity of positive control (1 nM E2). Data represent the mean ± standard error of the mean (*n* = 3). ^*∗*^*p* < 0.05 vs. the blank group; ^#^*p* < 0.05 vs. the E2 group.

To confirm the involvement of ERs in the enhancement of TMX-induced cell death using geraniol, we investigated the interactions between ER*α*/*β* and geraniol (Figure 7). Geraniol was the only compound that bounded to both ER*α* and ER*β*. Other constituents, such as citronellol, bounded with neither. Moreover, the ER agonist activity of geraniol was similar to that of *Geranium* oil. Despite their activities as ER agonists, coadministrating them with E2 indicated that both inhibited the ER agonist activity of E2. It is possible that geraniol competes with E2 as a partial ER agonist. These data suggest that geraniol is an essential compound of *Geranium* oil that is involved in the enhancement of TMX-induced cytotoxicity.

## 4. Discussion

GT1-7 cells possess a number of neuronal characteristics, including neurite extension and secretion of GnRH. They can also express receptors and neuron-specific proteins, including the E2 receptors (both ER*α* and ER*β* subtypes), microtubule-associated protein 2, tau protein, neurofilament, synaptophysin, GABA_A_ receptors, dopamine receptors, and L-type Ca^2+^ channels [15]. These properties make the GT1-7 cell line an excellent model system for investigating neurotoxicity and endocrine disruption [16, 17]. Previously, we developed a convenient *in vitro* assay to evaluate the neuroendocrine effects of essential oils [9]. We revealed that in GT1-7 cells, *Geranium* oil enhances the cell death induced by TMX, using this assay. Moreover, we identified the active compound involved in this activity: geraniol. Neither *Geranium* oil (Figure 1) nor geraniol (Figure 6) significantly reduced the cell viability of these cells in the absence of TMX. In a previous study, we confirmed that *Geranium* oil did not potentiate the stimuli responsible for inducing cell death, such as oxidative stress or administration of high-doses of zinc or aluminium [9]. In this study, we verified that reduced cell viability was caused by secondary effects that enhanced TMX cytotoxicity and not by *Geranium* oil (geraniol) directly.

TMX exerts antitumour effects via ERs. Previous studies have indicated that oestrogen is an important neurotrophic and neuroprotective factor [18]. In this study, TMX-induced cell death was alleviated by coadministrating the ER agonist E2 (Figure 3), suggesting that TMX might induce cell death by mediating the levels of ERs expressed in GT1-7 cells. *Geranium* oil contains geraniol. Howers et al. reported that geraniol was able to displace E2 from isolated ER*α* and ER*β*; however, it did not show oestrogenic or antioestrogenic activity in an oestrogen-responsive human cell line [19]. This report showed that a possible partial interaction between geraniol and ER was identified using molecular graphics models [19], which suggested that geraniol affected ER-mediated signal regulation by enhancing the effect of geraniol on TMX cytotoxicity. To test this hypothesis, we evaluated the ER activity of *Geranium* oil and geraniol using RCAS and found that both showed weak agonist activities on both ER*α* and ER*β*. Coadministration of these materials with E2 inhibited the agonist activities of E2 on ER*α* and ER*β*. These results suggest that geraniol, which is an essential compound involved in enhancing the effect of *Geranium* oil on TMX cytotoxicity, competes with E2 as a weak ER agonist.

Oestrogen enhances and maintains reproductive function and the function of the central nervous system (CNS). It acts on the brain to elicit reproductive behaviour, including solicitation of the opposite sex and mating behaviour in female mammals [20]. It also regulates synaptic transmission, synaptic plasticity, and adult neurogenesis and behaviour [21–23]. Moreover, numerous studies have demonstrated that oestradiol, the most active oestrogen, exerts trophic actions on neurones and glial cells, promotes neuronal survival, and decreases neurodegenerative damage caused by a large variety of neuronal insults [24]. In our previous report, we proved that *Geranium* oil protects GT1-7 cells from stress related to neurological disorders, such as oxidative stress or administration of high dose of zinc [9]. In the CNS, the blood-brain barrier (BBB) restricts the translocation of molecules; however, the nasal cavity has attracted attention as a route to directly access the CNS bypassing the BBB [25, 26]. A previous study showed that nasal administration of wheat germ agglutinin to rats resulted in high concentrations of it in the olfactory bulb located at the tip of the brain via the olfactory nerve, which is connected to the nasal mucosa. Other studies reported that intranasal administration of insulin-like growth factor-1 and interferon *β*1b to rats and monkeys quickly distributed them to the olfactory bulb and brain stem via the trigeminal nerve and olfactory nerve [27–29]. *Geranium* oil contains geraniol, which is said to exhibit oestrogenic activity [19]. It is possible that nasal inhalation of *Geranium* oil results in geraniol transport directly into the CNS, resulting in its associated activity.

The enhancing effect of *Geranium* oil on TMX cytotoxicity was also observed in another ER positive cell line such as human breast cancer cell line MCF-7 cells (Figure S1). TMX blocks oestrogen-neuronal binding on tumour surfaces to reduce tumour growth and proliferation in patients with breast cancer. In light of mounting evidence in the role of oestrogen in the maintenance of specific dimensions of cognitive function, these patients have a greater lifetime risk for cognitive impairment because of reduced oestrogen levels because of hormone therapy with TMX [30]. *Geranium* oil (and its component geraniol) is a relatively safe and nontoxic substance, when combined with TMX can potentially enhance its efficacy or reduce the required dosage. Moreover, we found that *Geranium* oil and its component geraniol have a weak oestrogenic effect. Shinohara et al. reported that exposure to *Geranium* oil increased the concentration of salivary oestrogen [31]. These findings suggest that *Geranium* may be useful for controlling the side effects of TMX caused by lower oestrogen levels. Therefore, the complementary use of essential oils or their active compounds with TMX can reduce the risk of cognitive impairment related to hormone therapy.

## 5. Conclusion

We showed that *Geranium* oil enhances TMX-induced cytotoxicity in GT1-7 cells as a partial ER agonist, with the essential compound associated with this effect being geraniol. Oestrogen is involved in the maintenance of reproductive function, brain function, and prevention of dementia. The nasal cavity has attracted attention as a noninvasive route for delivering substances directly to the CNS while bypassing the BBB. Nasally aspirated *Geranium* oil (and geraniol) might affect GnRH secretion through the ER, contributing to the treatment of neurological diseases, such as dementia. These results suggest that *Geranium* oil can be used for clinical applications, including supplementation with hormone therapy to alleviate the side effects of TMX in the CNS.

## Figures and Tables

**Table 1:** Amounts and standard deviation (*n* = 3) of the major constituents of *Geranium* oil.

Constituents	Amounts (%)	Concentration in 25 *μ*g/mL of *Geranium* (nM)
Citronellol	23.2 ± 0.45	37.2
Linalool	7.34 ± 0.07	11.9
Geraniol	2.38 ± 0.05	3.85
Citronellyl formate	7.29 ± 0.17	9.9
Isomenthone	0.44 ± 0.00	0.71

## Data Availability

The data that support the findings of this study are available from the corresponding author, Dai Mizuno, upon reasonable request.

## Supplementary Materials

Supplementary Materials
*Figure S1.* Effects of TMX and *Geranium* oil on MCF-7 cell viability. MCF cells were plated onto 96-well culture plates at a concentration of 5 × 10^4^ cells/well in 200 *μ*L of culture medium (Eagle's minimum essential medium with nonessential amino acids). Following 24 h incubation, cells were treated with 0.5 *μ*M TMX in the presence and absence of 25 *μ*g/mL of *Geranium* oil. After 24 h, the cell viability was analysed using WST-1 assay. Data represent the mean ± standard error of the mean (*n* = 6). ^*∗∗*^*p* < 0.01 vs. the TMX 0.5 *μ*M group.
