# Supplementary material for: Enhancing Cytotoxicity of Tamoxifen Using Geranium Oil
Source: Evid Based Complement Alternat Med. 2022 Mar 16;2022:8091339. doi: 10.1155/2022/8091339 (PMC8942665; doi:10.1155/2022/8091339)
Supplement: Supplementary Materials — Figure S1. Effects of TMX and Geranium oil on MCF-7 cell viability. MCF cells were plated onto 96-well culture plates at a concentration of 5 × 10^4^ cells/well in 200 μL of culture medium (Eagle's minimum essential medium with nonessential amino acids). Following 24 h incubation, cells were treated with 0.5 μM TMX in the presence and absence of 25 μg/mL of Geranium oil. After 24 h, the cell viability was analysed using WST-1 assay. Data represent the mean ± standard error of the mean (n = 6). ∗∗p < 0.01 vs. the TMX 0.5 μM group. [file 8091339.f1.docx]

**Figure S1**

**
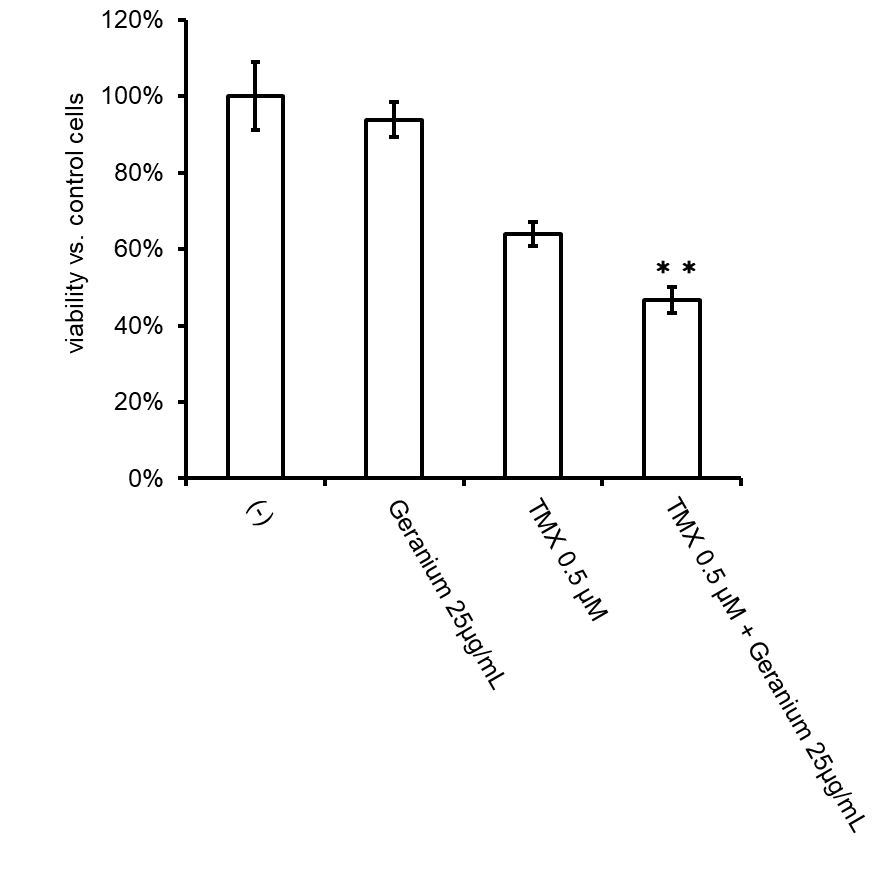
**

Figure S1: Effects of TMX and geranium oil on MCF-7 cell viability.

MCF cells were plated onto 96-well culture plates at a concentration of 5 ×10^4^ cells/well in 200 μL of culture medium (Eagle's minimum essential medium with non-essential amino acids). Following 24 h incubation, cells were treated with 0.5 μM TMX in the presence and absence of 25 µg/mL of geranium oil. After 24 h, the cell viability was analysed using WST-1 assay. Data represents the mean ± standard error of the mean (n = 6). **p < 0.01 vs. the TMX 0.5 μM group.
